# Nature-Based Hydrogels Combined with Nanoparticles for Bone Regeneration

**DOI:** 10.3390/jfb16090317

**Published:** 2025-08-30

**Authors:** Margarida Fernandes, Mónica Vieira, Daniela Peixoto, Natália M. Alves

**Affiliations:** 1 3B’s Research Group, I3Bs—Research Institute on Biomaterials, Biodegradable and Biomimetics, University of Minho, Headquarters of the European Institute of Excellence on Tissue Engineering and Regenerative Medicine, AvePark, Parque de Ciência e Tecnologia, Rua Ave 1, Edifício 1 (Sede), Barco, 4805-694 Guimarães, Portugal; 10230719@ess.ipp.pt; 2 ICVS/3B’S—PT Government Associate Laboratory, Braga, 4805-694 Guimarães, Portugal; 3 RISE-Health, Center for Translational Health and Medical Biotechnology Research (TBIO), ESS, Polytechnic of Porto, R. Dr. António Bernardino de Almeida, 400, 4200-072 Porto, Portugal; mav@ess.ipp.pt

**Keywords:** biomaterials, hydrogel systems, nanoparticles, bone

## Abstract

Bone is a calcified tissue composed of 60% inorganic compounds, 30% organic compounds, and 10% water. Bone exhibits an intrinsic regenerative capacity, enabling it to heal after fractures or adapt during growth. However, in cases of severe injury or extensive tissue loss, this regenerative capacity becomes insufficient, often necessitating bone graft surgeries using autografts or allografts. Conventional grafting approaches present several limitations, driving the development of alternative strategies in tissue engineering. The system of hydrogel–nanoparticles (NPs) represents a new class of biomaterials designed to combine the advantages of both materials while mitigating their drawbacks. This review focuses on a combination of nature-based hydrogels with different types of nanoparticles and discusses their potential applications in bone regeneration.

## 1. Introduction

Bone is a calcified tissue that is composed of 60% of inorganic compounds (hydroxyapatite), 10% water, and 30% of organic compounds (proteins) [1,2]. Its primary function includes providing support to the musculoskeletal system, protecting vital organs such as bone marrow and brain, maintaining mineral homeostasis, and serving as a source of calcium and bone marrow storage [3]. The cells present within the extracellular matrix (ECM) enable the tissue to self-regenerate and adapt as necessary [1].

It is a highly vascularized tissue with physiological functions such as detoxification, maintaining body structure, and acting as an endocrine organ to produce and sustain mineral substances [4,5]. One of the main characteristics of bone is its ability to regenerate in response to an injury or during growth [4]. However, this regenerative process may be impaired when the level of damage is significant [5].

**Figure 1:**
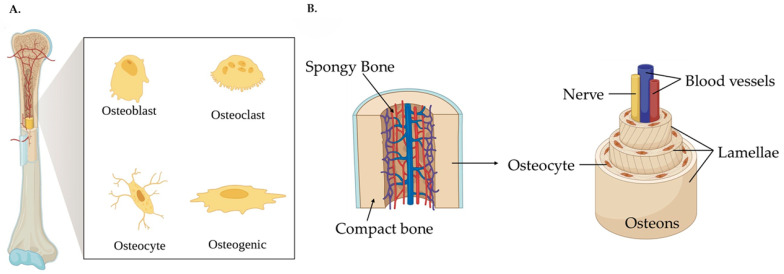
Structure and cellular composition of bone tissue: (**A**) overview of the major bone cell types; (**B**) structural morphology of the bone.

The four most important bone cells are osteoblasts, osteoclasts, osteogenic cells, and osteocytes (Figure 1A). Osteoblasts play a key role in creating new bone tissue by producing essential components of the ECM, such as collagen, proteoglycans, non-collagenous proteins, and cell adhesive proteins. Additionally, vesicles secreted by osteoblasts, which are localized in the ECM, create a nourishing environment for the nucleation of calcium phosphate to occur [3,6].

Osteoclasts are multi-nucleated cells provided by monocytes, responsible for the reabsorption of bone in response to local stimuli. This process is fundamental to maintain the calcium levels in the blood. Osteocytes are osteoblast cells surrounded by mineralized bone tissue, where their location and interconnectivity make them ideal for detecting and translating mechanical stress into a physiological response. Lastly, the osteogenic cells create a thin layer of bone tissue acting as a protective layer against osteoclast activity. In general, all types of cells are responsible for preserving and repairing bone tissue, so the supply of oxygen and nutrients must occur to ensure their survival [3].

Regarding the structural morphology of the bone tissue, presented in Figure 1B, the bone is divided into osteons, lamellae, collagen fibers, and fibrils. The collagen fibrils consist of collagen type I molecules of subnanometer size, separated by crystal plaques like hydroxyapatite and other impurities such as phosphoric acid, sodium, magnesium, and potassium. These fibrils self-assemble in packs and align in specific directions, forming collagen fibers with diameters ranging from 3 to 7 μm. The concentric assembly of these fibers forms osteons, a structure that, when involving the Haversian canals, forms the Haversian system [3].

The different types of compounds inside the bone can create two different types of bones: cortical and trabecular. The cortical bone is the denser external bone part due to the concentric distribution of the osteons. The trabecular bone consists of a more open network, where the free spaces are occupied by the blood vessels and the bone marrow [3,7].

## 2. Healing Process of Bone Fractures

The natural healing process of bone fractures is a complex physiological process involving diverse cell types and cellular responses. It is divided into two groups: primary or direct treatments of cortical fractures and indirect or secondary healing treatment [8].

**Figure 2:**
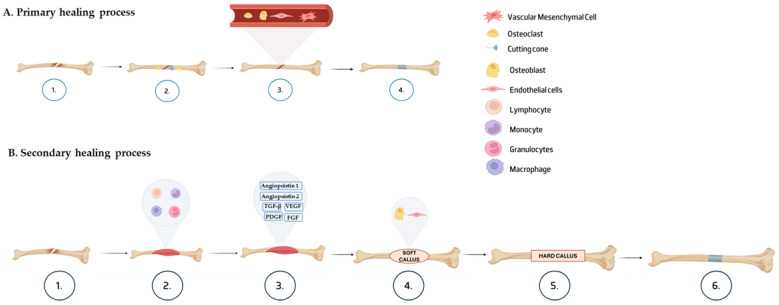
(**A**) Primary healing process: (1) bone fracture; (2) formation of cutting cone at the end of osteons that will cross the fracture site, creating cavities with the help of osteoclasts; (3) vessel creation with the presence of osteoclasts and endothelial and mesenchymal cells that will help bone regeneration; (4) final step, bone completely regenerated. (**B**) Secondary healing process: (1) bone fracture; (2) formation of the hematoma and infiltration of inflammatory cells; (3) release of TGF-β, PDGF, GFG, VEGF, and angiopoietins 1 and 2, which will help the angiogenesis process of creating new blood vessels; (4) formation of soft callus surrounding the wound; the mineralization process starts when it is stabilized; (5) hard callus formation; (6) regenerated bone.

The primary healing process, Figure 2A, occurs when there is an anatomic reduction of the fracture fragments and an interfragmentary reduction [8]. If these requirements are achieved, the bone can heal by remodeling the lamellar bone and Haversian channels [9]. This process is characterized by the fact that callus formation does not occur due to the lack of periosteal response. Instead, there is a cortex activity of trying to replace mechanical continuity by re-establishing a new Harversian system [8]. One side of the cortex must connect with another bone to establish mechanical and physical continuity. Cutting cones are formed at the end of the osteons, near the fracture site, crossing the fracture line, creating longitudinal cavities, via osteoclasts, which are then filled with bone matrix, via osteoblasts [9].

On the other hand, the secondary healing process (Figure 2B) is characterized by the formation of a hematoma and micromotion, relying on the formation and dynamic deconstruction of the wound site. It is divided into subsets based on the two types of bone formation that occur: intramembranous and endochondral. The process is distinguished by the involvement of four different structures, the cortex, periosteum, bone marrow, and external soft tissue [8]. This is the most frequent type of healing process [9].

The bone healing process is a multi-tasking procedure that requires the presence of multiple cells. It can be divided into three phases: inflammation, repair, and remodeling [10].

The healing typically starts with hematoma formation with the entrance of blood in the wound area that is reabsorbed through the hours. This is followed by the infiltration of inflammatory cells, such as macrophages, granulocytes, lymphocytes, and monocytes, to prevent infections and release growth factors and cytokines, initiating the coagulation process [8]. These cells are also responsible for eliminating necrotic tissue, inducing angiogenesis, and recruiting progenitor cells [10]. Degranulating platelets are also present in the clot, which may release transforming growth factor beta (TGF-beta) and platelet-derived growth factor (PDGF), a process that can trigger angiogenesis and chemotaxis. Additionally, bone morphogenetic proteins (BMPs) are also released from the bone matrix area, primarily expressed by the recruited stem cells to facilitate the ossification process, playing a role in the mesenchymal stem cell (MSC) differentiation [8]. The exposition of MSCs to osteogenic BMPs results in the augmentation of the expression of specific osteoblast markers, like alkaline phosphatase (ALP), osteocalcin, osteopontin, and connective tissue growth factor (CTGF) [11,12]. In this phase, the wound site contains high levels of pro-angiogenic factors, including fibroblast growth factor (FGF), vascular endothelial growth factor (VEGF), and angiopoietins 1 and 2. It is believed that angiopoietins have an important role in the beginning steps, while VEGF has an important role during the formation of endochondral bone [8].

In the repair phase, the progenitor cells, like MSCs and skeletal stem cells (SSCs), will have the potential to expand and undergo chondrogenic and osteogenic differentiation to form a fracture callus. The fracture space is linked by cartilaginous tissue, formed by chondrocytes, which become hypertrophic, and the cartilage is converted into bone. In these processes, cartilage absorption and chondrocyte apoptosis are crucial to allow the ossification by the invasion of progenitor cells. It is also suggested that some chondrocytes can directly differentiate into osteoblasts and promote bone formation [10].

The process of bone regeneration culminates in the bone remodeling stage, which involves the fusion of the hard callus from intramembrane ossification and the soft callus from endochondral ossification. Bone remodeling is essential for the bone to maintain its ability to bear weight, and it entails the use of osteoclasts to reabsorb the mineralized bone in the hard and soft callus. During this reabsorption process, osteoblasts deposit new bone, forming lamellar bone with the ability to support weight, ultimately resulting in the restoration of the cortical structure [8]. This stage can take months to years to be fully completed [9].

## 3. Bone Regeneration Strategies

Millions of people suffer from bone defects due to traumas, diseases, congenital malformations, and cancer. One study in 2019 showed that approximately 178 million persons (53% men and 47% women) suffer from bone fractures, for which worldwide, close to 2.2 million bone grafting surgeries are performed every year [4], making it the second most transplanted tissue in the world [13].

The traditional treatment for patients with low or incomplete bone healing of fractures is the use of bone grafts, using either autografts or allografts. However, there are some limitations when using bone grafts [13,14,15,16].

The use of bone from the patient, autograft bone, presents diverse advantages, mainly due to being immunocompatible with the patient, a source rich in BMPs, fibroblast growth factors, VEGF, growth factors derived from platelets, and insulin-like growth factors. They present osteogenic, osteoinductive, and osteoconductive properties, with reduced risk of rejection and transmission of diseases. Nevertheless, it is necessary to be taken out of places that do not support large quantities of weight, and there is also a limited graft supply, donor site morbidity, and the possible development of chronic pain and infections. The techniques used to remove the autologous bones significantly affect cell viability, bone integration, and their regenerative capacity [17].

The allografts are derived from live donors or cadavers. They are considered a source of collagen type I and BMPs, which give them osteoinductive properties. However, as they are not from the patient, they possess different genetic compositions leading to concerns about immune rejection, blood compatibility, and disease transmission [18]. Compared to the autografts, they present a higher failure rate due to their immunogenicity, possibly being rejected due to antigen activation [17].

Since traditional treatments have diverse disadvantages, tissue engineering has emerged as a viable approach for treating high-grade fractures when the self-repair capacity is insufficient to repair the damage by creating new devices/materials for regeneration [5]. In particular, this review will focus on the use of natural-origin hydrogels combined with nanoparticles for bone regeneration applications.

Hydrogels have gained more attention from bone tissue engineering (BTE) because of the possibility of designing biomaterials where the matrix is charged with molecules to induce osteogenesis [4] and for presenting a porous structure similar to the ECM, having good biocompatibility, and having a smooth structure that reduces the inflammatory response of the cells in the surrounding tissue [13,19].

Hydrogels are a macromolecular network capable of withholding a large amount of water, where 99% of their weight can be water, making them highly compatible with biological environments abundant in water, such as the human body [20], a capability provided by the hydrophilic groups in the structure [21]. The gelation process could occur before being introduced in the biological system or in situ after administration, and such processes could be triggered by different stimuli in the human body, such as temperature, pH, or ionic charges [20].

**Figure 3:**
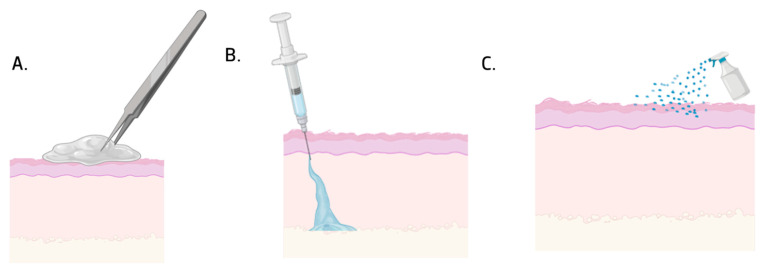
Types of hydrogels: (**A**) implantable; (**B**) injectable; (**C**) sprayable.

The interaction between hydrogels and cells is a complex and dynamic process, impacting the physiology of the tissue (e.g. proliferation, migration, differentiation, and cell spreading) and pathological processes (e.g. cell apoptosis, fibrosis, and immunologic rejection). In general, when exposed to the hydrogel matrix, cells respond to the hydrogel’s physical and chemical characteristics, porous size, stiffness, viscoelasticity, degradability, and microarchitecture. According to these properties, cells will adapt to regulate their biology and homeostasis [22]. Hydrogels can be implantable, sprayable, and injectable, as shown in Figure 3 [20].

Implantable hydrogels are 3D hydrogels with sizes typically ranging from millimeters to centimeters, where the polymeric chains are cross-linked through physical and chemical interactions. Chemical cross-linking results in permanent junctions within the polymeric network, and physical cross-linking occurs due to the interconnection of polymeric chains or through physical interactions such as hydrogen bonding, hydrophobic interactions, and ionic bonding. They are normally implanted surgically or in direct connection with the human body [23].

Sprayable hydrogels are highly promising because of the number of advantages when compared with traditional hydrogels. They are easily sprayed on irregular surfaces, creating thin and clear films, giving better coverage and conformability in the target area, providing a moist environment that supports the formation of new tissue. They can be modified to retain a large variety of physical, chemical, and biological properties, such as stiffness, porosity, bioactivity, and biodegradability properties, modified for each type of tissue. For their application to be effective, the materials must exhibit shear-tinning properties, which allow them to be delivered in a “sol-like” state and then change in situ to a gel form after the cross-linking process [24].

Lastly, injectable hydrogels are hydrogels with physical–chemical properties that allow them to be injected into the human body in a liquid state and gelify after contact with the interior of the body [25,26]. They undergo gelling in situ to permit irregular spaces in wounds to be filled, causing less damage [26]. They have been highly used in biomedical applications because of their biocompatibility, biodegradable capacity, and reduced secondary effects [27].

Despite their advantages, hydrogels have some limitations when applied to bone regeneration. They may trigger inflammatory and immune-mediated responses, as well as local and systemic adverse reactions. This material exhibits reduced mechanical properties, elasticity, stability, and toughness, along with rapid degradation. Moreover, hydrogels are unable to replicate the complexity of the biological environment, showing limited durability at the injury site, because they cannot bear the same load that natural tissue normally supports [25,28,29,30,31,32,33,34].

The use of nanomaterials has been adopted as a strategy for bone regeneration. Nanoparticles (NPs) can be combined with different biomaterials, offering adjustable mechanical strength and stimulating the differentiation of stem cells into osteoblasts, and in some cases, NPs on their own can enhance osteogenesis [35]. In the last years, the possibility of incorporating NPs in hydrogels for applications in tissue engineering, drug delivery, wound treatment, immune modulation, and detoxification has been evaluated [36]. For example, the utilization of NPs as an antimicrobial agent is appealing due to their small size, large surface area, and distinctive physico-chemical properties, allowing for improved drug bioavailability and target delivery [36].

For bone regeneration applications, nanoparticles (NPs) also present certain limitations, such as low osteoinductive potential and poor mechanical strength. Additionally, their biodistribution, size uniformity, and tendency to agglomerate within tissues are challenging to control, which raises the risk of systemic toxicity and inflammation [37,38,39,40].

The NP–hydrogel systems represent a new class of biomaterials drawn to combine the advantageous properties of every material type while mitigating their drawbacks. These systems can be utilized in different biomedical applications, including tissue engineering, drug delivery, immune modulation, antibacterial resistance reduction, and wound treatment [36]. The incorporation of NPs in the hydrogel enhances the mechanical force, stiffness, swelling, stability, and biocompatibility. It also increases the antibacterial properties, bioactivity, and biological properties with the improvement of cell adhesion, osteodifferentiation, and stimulation of new bone formation [41,42,43,44,45,46,47]

This system promotes cell viability, proliferation, and differentiation because it provides a microenvironment similar to the ECM regarding its mechanical properties and architecture. The presence of NPs allows for higher cell adhesion by providing a higher surface area, higher roughness, and hydrophilicity. The release of ions from the NPs enhances the cellular response, permitting their adhesion, proliferation, and differentiation, also augmenting the expression of genes associated with bone [48,49,50,51,52].

Next, we present an overview of natural polymers and their combinations with various nanoparticles to create hydrogel systems with enhanced properties for bone regeneration.

## 4. Nature-Based Hydrogels Combined with Nanoparticles

### 4.1. Collagen

Collagen is a glycoprotein obtained from different types of animals. It is one of the principal compounds of the ECM, being the most abundant protein in the human body [53], essential to bone tissue, cartilage, tendons, and blood vessels [54]. It is the bone’s principal compound, making it ideal for developing new scaffolds for bone applications. It is biocompatible, bioactive, and rich in binding sites on the surface, stimulating cell adhesion, proliferation, and differentiation [53].

Montalbano et al. [55] and Borciani et al. [56] produced collagen-based hydrogels with bioglass nanoparticles for bone regeneration applications. They have been strongly studied due to their osteoconductivity, osteostimulation, and degradation rate. They have the ability to form strong interfacial connections between the bone and the material, creating a hydroxyapatite layer and releasing ions that induce bone formation [57,58].

Montalbano et al. [55] studied the possibility of applying collagen hydrogels with mesoporous bioglass nanoparticles with 4% strontium (MBG_Sr4%) in bone tissue engineering. It was observed that the incorporation of NPs influenced osteoblast activity and osteogenic differentiation. These hydrogels were also viable for MG-63 cells and allowed for cell adhesion.

**Figure 4:**
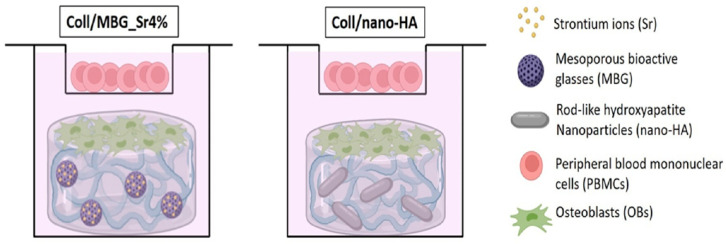
Co-culture set-up. Reprinted from [56].

Borciani et al. [56] also proposed the use of collagen-based hydrogels with mesoporous bioglass nanoparticles with 4% strontium (Coll/MBG_Sr4%) or with nanohydroxyapatite (Coll/nano-HA) (Figure 4). After 21 days, they observed that the hydrogels with MBG_Sr4% exhibited higher osteoblast viability compared to Coll/nano-HA. For PBMCs, the Coll/nano-HA hydrogel showed a more significant increase in cell viability, although it declined over time in both hydrogels. Live/Dead staining reflects the previously reported results. ALP activity was also measured, registering an increase in both hydrogels after 14 days, with higher expression in the Coll/MBG_Sr4%. 

### 4.2. Silk Fibroin

Silk fibroin is a natural material renowned for its high tensile strength and stiffness [59]. Derived from silkworms, it has become a focal point in the field of biomaterials due to its biocompatibility, excellent mechanical properties, low degradation rate, non-toxicity, and non-immunogenic nature [59,60].

Silica, nanohydroxyapatite, silver, and gold nanoparticles have been gaining more attention for bone treatments due to their important characteristics. For silica NPs, it has been described that they activate, both in vitro and in vivo, the formation of bone cells like osteoblasts while inhibiting bone reabsorption and reducing osteoclast activity [61]. Nanohydroxyapatite has the advantages of small particles, high surface area, and high surface energy, which promote cell adhesion and proliferation, leading to strong osteoconduction and biomineralization capacity. It is commonly used in bone applications due to its chemical similarities with inorganic compounds of bone matrix and because of its osteoinduction, osteoconductive, and osteointegration capacity [62,63,64]. Regarding silver and gold nanoparticles, they have gained attention in biomedical applications mainly due to their antimicrobial activity against a series of bacteria [65,66].

**Figure 5:**
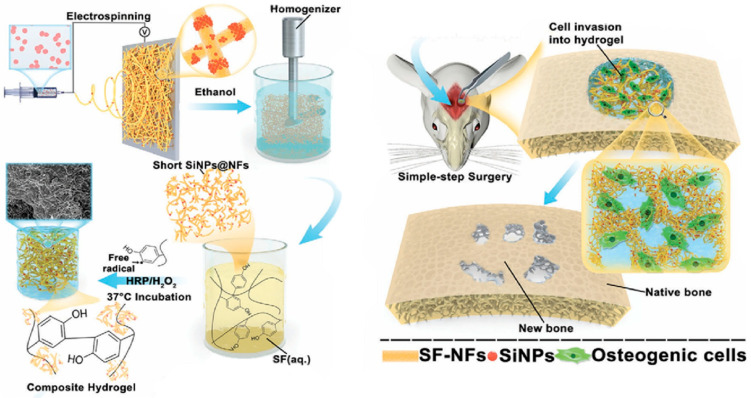
Representation of the composite hydrogel. Reprinted with permission from Ref. [67]. Copyright 2021 John Wiley and Sons.

Cheng et al. [67] studied silk fibroin (SF) hydrogel with silica nanoparticles (SiNPs@NFs) (Figure 5) for bone applications. They tested four different SiNP concentrations: 0%, 1%, 3%, and 5%. The SiNPs@NFs5%-SF showed higher viability than the other concentrations; nevertheless, all concentrations demonstrated better viability than the control. For the in vitro tests, an MC3T3-E1 cell line was used, and it was observed that the hydrogels with 5% SiNPs presented enhanced adhesion, proliferation, and osteogenic differentiation of preosteoblasts. In vivo studies in a cranial fracture model in rats revealed that the group with 5% SiNPs formed bone more rapidly than the other groups. 

Additionally, Ribeiro et al. [62] produced silk fibroin hydrogels with hydroxyapatite nanoparticles (nHA) at concentrations of 0 wt%, 10 wt%, and 15 wt%. Some of the hydrogels were frozen at −20 °C to assess whether this would affect their characteristics. In vitro studies, using osteoblast-like cells (MG63 cell line), were conducted with 15 wt% nHA hydrogels because those with 10 wt% exhibited lower compression moduli due to their higher porosity. The metabolic activity of both frozen and unfrozen hydrogels increased over time, with higher levels observed in the frozen samples. In terms of ALP activity, the increase was more pronounced in the frozen hydrogels. Compared to silk fibroin hydrogels, the composite hydrogels with nHA were more favorable for bone tissue engineering applications due to improved cell metabolism and ALP activity.

In 2017, Ribeiro et al. [65] produced silk fibroin hydrogels with nanohydroxyapatite, which were combined with either silver or gold nanoparticles. Gold (AuNPs) and silver NPs (AgNPs) were added at different concentrations: 0%, 0.1%, 0.5%, and 1%. In vitro studies demonstrated that when added at concentrations higher than 0.5% AgNPs, the hydrogels were toxic to MG63 cells. Regarding the concentrations of AuNPs used, no toxicity was observed in the same cells, with similar viability values across all three concentrations tested. Both types of hydrogels supported the adhesion and spreading of osteoblasts.

In another study, Daneshvan et al. [68] produced pluronic grafted silk fibroin hydrogels incorporating hydroxyapatite nanoparticles (nHA) at three different concentrations: 5%, 10%, and 20%. The addition of these nHA promoted osteogenesis and enhanced cytocompatibility compared to the hydrogels without nHA; however, the percentage of 20% of nHA reduced cell proliferation. The addition of 10% of nHA showed better results with higher values of ALP activity, more calcium deposition, and the highest cell viability of MG-63 cells. These results indicated that the use of concentrations higher than 20% nHA was toxic to MG-63 cells. 

### 4.3. Hyaluronic Acid

Hyaluronic acid is a hydrophilic ECM compound [22] that is widely distributed throughout various tissues [34]. It features binding sites that assist in cell migration and differentiation [22], enhancing cell survival and supporting angiogenesis [34]. It has a remarkable ability to retain water in wound areas, preventing dryness and promoting rapid healing [21]. Its hydrophilic nature promotes the adsorption of BMPs and growth factors that are involved in bone formation and regeneration. The presence of these growth factors can enhance the osteogenic differentiation of MSCs and promote the formation of new bone [69].

Bisphosphonate–magnesium NPs could be used as bone regeneration material because they present a variety of bioactive and excellent ligation sites with affinity for multivalent cations like Mg^2+^. The release of the cations Mg^2+^ increases cell adhesion and spreading, possibly promoting osteogenic differentiation [70].

**Figure 6:**
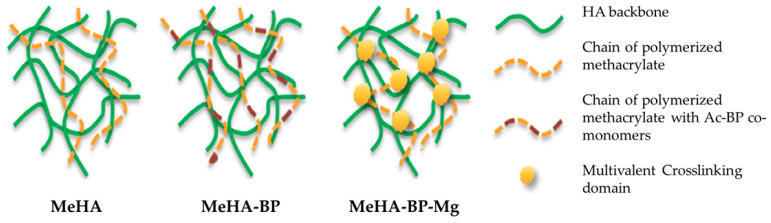
Scheme representation of the different hydrogels. Reprinted with permission from Ref. [70]. Copyright 2017 Elsevier.

Zhang et al. [70] produced hydrogels based on methacrylated hyaluronic acid (MeHA) and acrylate biphosphate nanoparticles (Ac-BP), without and with magnesium (BP-Mg) (Figure 6). They produced three different hydrogels, one without BP (MeHA), one without magnesium (MeHA+BP), and one with magnesium (MeHA+BP-Mg). The formed hydrogels facilitated cell adhesion and spreading, promoting hydrogel mineralization and osteogenic differentiation of hMSCs and in situ bone regeneration. When comparing the three types of hydrogels, it was concluded that the MeHA+BP-Mg system presented higher expression of osteogenic markers and a more elevated number of cells per mm^2^. In vivo studies in a calvarial defect model in rats showed that this hydrogel formed more new bone compared to other hydrogels. These results revealed that the incorporation of Mg in the BP allowed bone regeneration to occur more rapidly.

### 4.4. Fibrin

Fibrin is an important protein in the construction of the ECM [34] with an important role in orthopedic surgeries, acting as a sealant and adhesive agent, promoting angiogenesis and osteogenesis, while having the capacity to retain various cells [71].

Fibrin-based hydrogels are utilized in tissue engineering and clinical applications because of their excellent biocompatibility, elasticity, adhesive properties, and the fact that their degradation products are non-toxic [72].

Graphite oxide NPs are obtained by the reaction of graphite and oxidation reagents using the modified Hummers’ method. Due to their properties of easy dispersion in aqueous solutions, degradability, mechanical stability, and elevated surface area, these materials are promising for bone regeneration. They promote osteogenic cells’ adhesion and proliferation [71,73].

Pathmanapan et al. [71] produced fibrin hydrogels with nanohydroxyapatite and graphene oxide nanoparticles (GO). These hydrogels showed enhanced viability in NIH-3T3 and MG-63 cells at 40 mg/mL. They also improved biocompatibility, augmented ALP activity, and increased osteogenic differentiation. In vivo studies showed progress in the healing treatment with the composite formed. In fact, these hydrogels created a microenvironment conducive to the intra-chondral formation of MSCs, facilitating the effective formation of new bone tissue through indirect ossification. So, these hydrogels hold great potential for bone regeneration applications. 

### 4.5. Alginate

Alginate is a bioinert material [22] found in the cellular wall of the brown algae [34]. It is widely used in the biomedical field due to its biocompatibility and bioabsorbable properties [21]. The material has a simple gelation process and is a cross-linkable, injectable polymer that can be easily functionalized. Alginate is resistant to acidic conditions and carries a negative charge. However, it has low mechanical properties, uncontrolled degradation kinetics, and challenges with sterilization and handling, which can lead to the leaching of the entrapped drugs [53].

Due to their ability to absorb large amounts of water, alginate-based hydrogels can effectively remove secretions from wound sites, controlling bacterial growth [21]. This type of hydrogel exhibits tunable viscoelasticity and stress relaxation time, which enhances the cell contraction force and improves interaction with cell ligands [22].

Selenium biphasic calcium NPs (Se-BCP) are advantageous for tissue engineering applications due to their controlled bioactivity, progressive degradation, promoted osteoconductivity, and presented equilibrium between reabsorption/solubilization. These characteristics make them ideal for promoting bone growth. Selenium is an essential element for all living organisms, critical for antioxidant defense. It can support immunologic surveillance, cell proliferation, and differentiation. These Se-BCP present osteoconductive and immunomodulation actions, crucial for bone regeneration [74].

**Figure 7:**
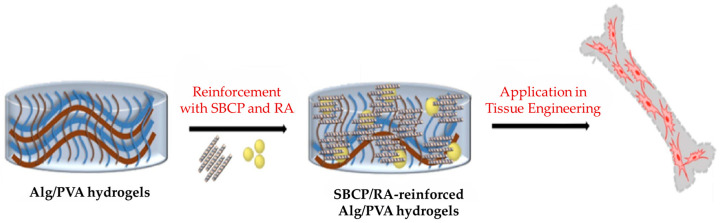
Alginate/PVA with SBVP/RA hydrogels. Reprinted with permission from Ref. [74]. Copyright 2024 Elsevier.

Singhmar et al. [74] proposed hydrogels based on alginate (Alg) and polyvinyl alcohol (PVA) combined with Se-BCP (Figure 7). Four different compositions were studied: a control formed with Alg and PVA, a second solution formed with BCP, a third formed with Se-BCP, and a fourth formed with Se-BCP + 0.1% retinoic acid (RA), a compound that stimulates osteogenic differentiation, proliferation, and migration. All hydrogels produced were bioactive, allowing for the formation of hydroxyapatite after being immersed for 14 days in fetal bovine serum. However, differences were observed in the viability and proliferation of MC3T3-E1 cells. The control group showed the lowest viability and proliferation, followed by the hydrogels + BCP and then the hydrogels + Se-BCP, with the hydrogels + Se-BCP + RA exhibiting the highest viability and proliferation. It was concluded that the addition of Se-BCP enhanced viability and cell proliferation and that the addition of RA augments these characteristics.

Also, Barros et al. [64] produced alginate hydrogels incorporating nanohydroxyapatite (nano-HA) at different concentrations of 0 wt%, 30 wt%, 50 wt%, and 70 wt%. The hydrogels containing 30% wt% nano-HA supported enhanced the in vitro proliferation of osteoblasts and human mesenchymal stem cells (hMSCs), as well as increased metabolic activity and DNA content compared to the other concentrations. Ex vivo studies further showed collagenase deposition and formation of trabecular bone.

### 4.6. Chitosan

Chitosan is a natural polysaccharide derived from chitin [53], which can be produced from crustaceans, insects, and some microorganisms, through various enzymatic and chemical processes [4]. It has been utilized in wound treatment due to its antibacterial properties [21], biocompatibility, biodegradation, non-toxicity, and non-antigenic properties. Additionally, it is inexpensive [53] and positively charged, allowing it to better absorb anionic metallic drugs [21]. Some studies also reported its osteoconductive properties, which can promote the formation of new bone [4]. However, chitosan has limitations, including both low mechanical strength and stability, immunogenicity, and a prolonged time for bone formation compared to other polymers [53].

Magnesium oxide NPs can be used in bone regeneration applications due to their biodegradability, minimal toxicity, biocompatibility, antimicrobial, and antioxidant properties. The incorporation of these NPs into scaffold structures enhances their mechanical, osteogenic, angiogenic, and osteoconductivity properties, facilitating bone tissue growth [75,76,77].

Chen et al. [78] produced methacryloyl chitosan with 3-phosphonopropionic acid (CSMAP) hydrogels, with magnesium oxide nanoparticles (MgO NPs) incorporated at different concentrations (0.5%, 2.5%, 5%, and 10%). The incorporation of MgO NPs enhanced the mechanical properties and stability of the hydrogels and conferred osteogenic capacity, promoting in vitro mineralization when in contact with supersaturated calcium phosphate solutions. The authors also demonstrated that these hydrogels promoted osteogenic differentiation and mineralization of MC3T3-E1 cells, with increased capacity to promote the formation of new bone in in vivo studies.

Also, Chen et al. [75] in 2022 produced methacryloyl chitosan with phosphocreatine (CSMP) hydrogels incorporating MgO NPs at concentrations of 0, 0.5, 2.5, 5, and 10 mg/mL. The different types of hydrogels produced promoted the deposition of calcium phosphate, cell proliferation of MC3T3-E1, osteogenic differentiation, mineralization, and a high expression of osteogenic genes (BSP and OPN). In vivo studies in a cranial defects model in rats showed that using CSMP-MgO at a concentration of 5 mg/mL promotes better bone regeneration with a higher reduction of fracture site size. The differences between the two works developed by Chen et al. [75] are the hydrogels’ compositions, where the second study shows an optimization of the previous works.

### 4.7. Gelatin

Gelatin is a protein substance derived from the hydrolysis of collagen [79]. It is biocompatible, biodegradable, has favorable cell recognition properties, and can be easily modified to create new structures [53]. Chemically, gelatin contains a low quantity of aromatic groups, resulting in low immunogenicity [80]. It is composed of arginine–glycine–aspartic acid peptides that promote cell adhesion, proliferation, and differentiation, making it effective for stimulating ECM [80]. However, it has a low stability, which typically requires chemical cross-linking [53].

Heo et al. [81] studied the possibility of applying gold nanoparticle (AuNP)–methacrylated gelatin (GelMA) composite hydrogels for bone regeneration. Three different compositions were produced with varying AuNP concentrations: 0.014 µg/μL, 0.071 µg/μL, and 0.2 µg/μL. To compare the role of AuNPs with a growth factor that is known to help bone grow and regenerate (BMP-2), a group was formed, consisting of 10 mg of BMP-2 and 10% GelMA. The results obtained showed that the hydrogels with AuNPs promoted higher ALP and osteogenic differentiation of human adipose-derived stem cells (ADSCs) in a similar manner to the ones with BMP-2. In vivo studies using a rabbit skull defect model demonstrated that hydrogels containing higher concentrations of gold nanoparticles (AuNPs) yielded more favorable outcomes, including enhanced new bone formation and a reduction in wound size. These results suggest that AuNPs can be applied as effective osteogenic promoters by stimulating osteogenic differentiation. 

**Figure 8:**
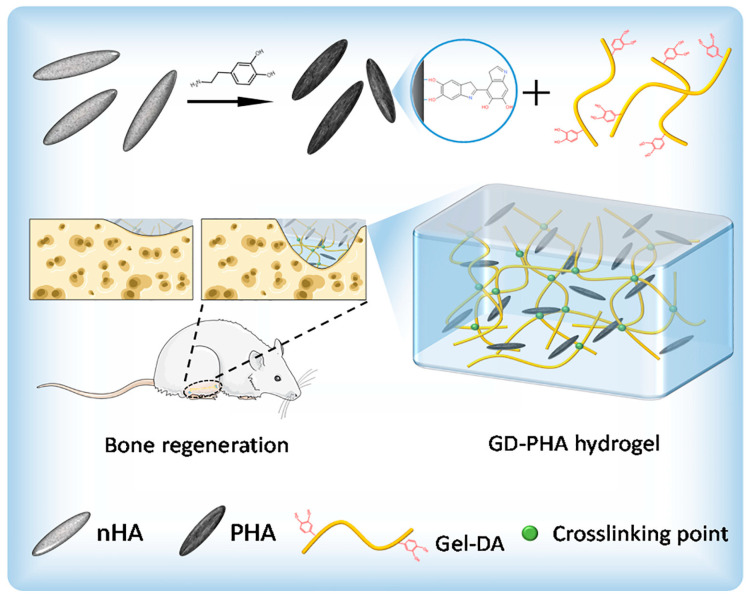
Schematic illustration of the synthesis of the GD-PHA hydrogel. Reprinted with permission from Ref. [63]. Copyright 2023 Elsevier.

Another study by Ma et al. [63] analyzed the possibility of using hydrogels of gelatin with catechol groups incorporating nanohydroxyapatite (nano-HA) and nano-HA modified with polydopamine (PHA) (Figure 8). Chemical modifications of polymers like gelatin with catechol groups allow for the development of a system with enhanced capacity to adhere to wet environment. These hydrogels exhibited cytocompatibility with MC3T3-E1 cells, while those incorporating both types of nanoparticles showed increased cell viability, with the highest levels observed in the hydrogels containing PHA. In vivo studies using a femoral defect in the rat model were performed to evaluate the formation of new bone. It was observed that the hydrogel that incorporated nano-HA modified with PHA significantly accelerated new bone formation.

### 4.8. Gellan Gum

Gellan gum (GG) is a water-soluble polysaccharide produced by the fermentation of *Sphingomonas paucimobilis* bacteria. It presents structural and biological properties similar to those of the extracellular matrix [82]. It is a material that can be used in composite development due to its biocompatibility, biodegradability and injectability. GG-based hydrogels have been utilized in various applications within the tissue engineering field, allowing for the incorporation of organic and inorganic compounds [82]. For bone applications, it can be reinforced with diverse materials such as bioglass nanoparticles or nanohydroxyapatite [83].

Liu et al. [84] produced GG hydrogels with 1% *w*/*w* GG and 2.5 mM MgSO_4_ and with different nanohydroxyapatite (nano-HA) concentrations: 0%, 2.5%, 7.5%, and 10%. Regarding the cell viability of these hydrogels, the ones with 5% nano-HA presented the highest cell viability for BMSCs. In vitro studies with this condition showed that the hydrogel did not inhibit cell proliferation or augment apoptosis. It exhibited good cell adhesion and showed an increase in the expression of osteogenic genes, induced osteogenic differentiation, and maturation of the extracellular matrix. In vivo studies were conducted in a rat cranial bone defect model and showed that the GG/5%nHA/MgSO_4_ hydrogel stimulated vascularization and promoted new bone formation, with a more reduced wound site after 8 weeks.

Douglas et al. [85] produced GG-based hydrogels incorporating bioglass nanoparticles (BGNPs) with three different SiO_2_:CaO:Na_2_O:P_2_O_5_ formulations: A—40:54:0:6, B—80:16:0:4, and C—46:27:24:3. Before adding the BGNPs into the hydrogel matrix, they were mixed with MgCl_2_ and then inserted into the hydrogel with the solution at 40 °C. In the end, the hydrogels presented 0.7% *w*/*v* GG and 1% *w*/*v* for the three types of BGNPs, and these samples were compared with a control solution formed with GG/MgCl_2_. The produced hydrogels were bioactive, forming hydroxyapatite crystals after 21 days immersed in SBF, where GG-A presented higher antibacterial activity against Methicillin-resistant Staphylococcus aureus (MRSA) than the other systems. However, the GG-C hydrogels were the ones with better biological results; namely, they enhanced MG-63 cell adhesion and osteogenic differentiation markers (ALP). All the composite hydrogels supported the rat mesenchymal stem cells’ (rMSCs) growth.

## 5. Polymer Combination

Beyond being applied individually with nanoparticles, some researchers studied the possibility of conjugating more than one natural polymer with nanoparticles for bone applications.

For example, both Shahrebabaki et al. [86] and Chavez-Granados et al. [87] investigated the potential application of alginate–gelatin (Alg-Gel) hydrogels combined with nanoparticles (NPs) for bone applications.

**Figure 9:**
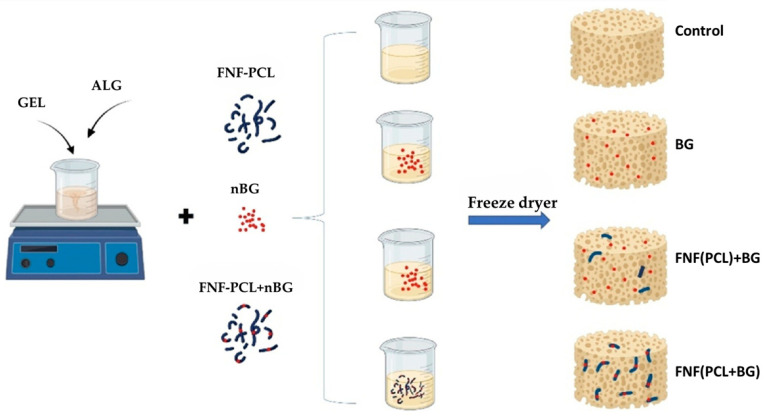
Schematic representation of the hydrogel formation. Reprinted with permission from Ref. [86]. Copyright 2024 Elsevier.

Shahrebabaki et al. [86] produced Alg-Gel hydrogels with bioactive glass nanoparticles (BGNPs) and fragmented nanofibers of polycaprolactone (FNF(PCL)). Four different compositions were analyzed: Alg-Gel (control), Alg-Gel + BG, and Alg-Gel + FNF(PCL) with BG outside the fibers’ matrix (Alg-Gel + FNF(PCL) + BG) or inside the fibers’ matrix (Alg-Gel + FNF(PCL + BG)) (Figure 9). It was observed that the presence of FNF(PCL) facilitated the adhesion, growth, and proliferation of MG-63 cells. When comparing the two types of hydrogels with FNF(PCL), FNF(PCL) + BG and FNF(PCL + BG), it was found that the Alg-Gel + FNF(PCL) + BG allowed for enhanced cell differentiation and exhibited lower toxicity, suggesting that the presence of BGNPs within the matrix produced better results than when it was included in the fibers. 

In the study developed by Chavez-Granados et al. [87], they incorporated silver NPs (AgNPs) into the Alg-Gel hydrogels. Before adding the AgNPs into the hydrogels, the cell viability was evaluated using different concentrations of AgNPs. It was found that they were toxic to human exfoliated deciduous teeth cells (SHEDs) at concentrations superior to 269.6 µg/mL. A concentration of 4 µg/mL was selected to be incorporated into the hydrogels for the in vitro tests, which was the one corresponding to the highest viability. The composite hydrogels demonstrated cell viability when compared to the hydrogels without AgNPs, promoting osteogenic differentiation, as shown by the increase in ALP expression. 

Selenium NPs have emerged as a promising material for bone regeneration due to their anti-inflammatory, antioxidant, and immune-modulator properties. They can enhance bone remodulation and support cell adhesion and proliferation, matrix mineralization, and osteogenic differentiation [88,89].

**Figure 10:**
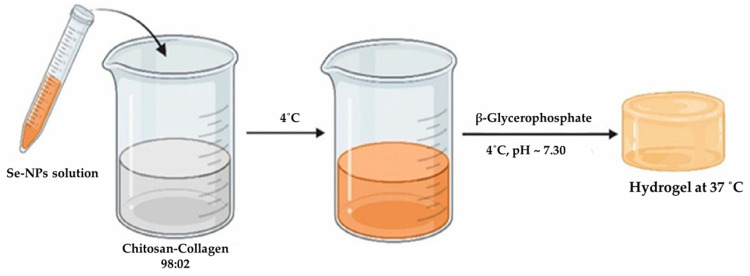
Synthesis of Se-doped hydrogel. Adapted from [88].

In the case of Alajmi et al. [88] and Kaur et al. [90], both produced collagen–chitosan (Coll-Chi) hydrogels with NPs (Figure 10). Alajmi et al. incorporated selenium nanoparticles (SeNPs) into the Coll-Chi hydrogels. They analyzed three different compositions: a control with just collagen and chitosan and two solutions with a concentration of 0.575 mM and 1.154 mM of SeNPs, respectively.

Regarding MSC proliferation, viability, and osteogenic differentiation, the samples with SeNPs presented better values than the control group. However, the results obtained were concentration dependent. Although both SeNP formulations allowed for cell proliferation and osteogenic differentiation, the samples with 2.56 mM affected negatively cell proliferation and cell viability, with results lower than the group with 0.575 mM SeNPs [88].

The possibility of applying carbon nanotubes in biomedical applications has been studied due to their elevated strength, elasticity, fatigue resistance, and antimicrobial activity. They can have a positive effect not only by stimulating cell adhesion but also by regulating cell morphology and accelerating stem cell differentiation, osteoblast differentiation, and apatite mineralization [90,91,92].

Regarding Kaur et al. [90], they produced Coll-Chi hydrogels with carboxylated single-walled carbon nanotubes (COOH-SWCNTs) with concentrations of 0.5, 1, 3, and 5 w/t% NPs. The addition of COOH-SWCNTs on the hydrogels enhanced MC3T3-E1 cell proliferation; however, the groups with higher concentrations showed lower proliferation, and the groups with 0.5% of COOH-SWCNTs were the ones with both higher proliferation and DNA concentration throughout time. Comparing the control group (without COOH-SWCNTs) with the hydrogels containing 0.5% and 5% COOH-SWCNTs, an increase in the expression of early osteogenic genes (RUNX2) and late osteogenic expression genes (OCN) was observed. This expression was reduced in the control group and more elevated in the hydrogels with COOH-SWCNTs, where the ones with 5% COOH-SWCNTs had a higher expression. It also evaluated the expression of calcium and ALP throughout the 14 days; the hydrogels with higher COOH-SWCNT concentrations were the ones with more ALP activity and more calcium deposition.

Graphene is a material that possesses excellent electrochemical properties, high thermal and electrical conductivities, infrared absorption, impermeability to gases, a high surface area, strong mechanical strength, porous structure, biocompatibility, bio-adhesion, and antibacterial activity. These properties make it suitable for various applications in fields such as electronics and biomedicine [93,94].

Seifi et al. [93], Wang et al. [95], Hia et al. [96], and Kasi et al. [97] produced hydrogels of chitosan combined with other natural polymers and NPs for bone regeneration applications.

Seifi et al. [93] produced chitosan (Chi) + alginate (Alg) + polyvinyl alcohol (PVA) filled with either carbon nanotubes (CNTs) or graphene nanoplatelets (GNP), respectively. They formed five different hydrogels composed of PVA, CS, and Alg with different ratios (4:6:4, 4:6:6, 4:6:8, and 4:6:10), with 1% (*w*/*w*) NPs and a control without NPs. The hydrogels formed presented values for the Young’s modulus, swelling, and degradation that were favorable for bone tissue engineering, as hydrogels with excessive degradation values would not support bone and cartilage repair. The hydrogels formed with the ratio 4:6:6 were selected for the in vitro tests because they presented better mechanical properties, swelling ratios, and degradability. The cell viability of MG63 cells increased in all hydrogels, and the addition of NPs further enhanced this increase. However, the hydrogels with CNTs were the ones that presented the highest percentages of cell viability after 7 days.

**Figure 11:**
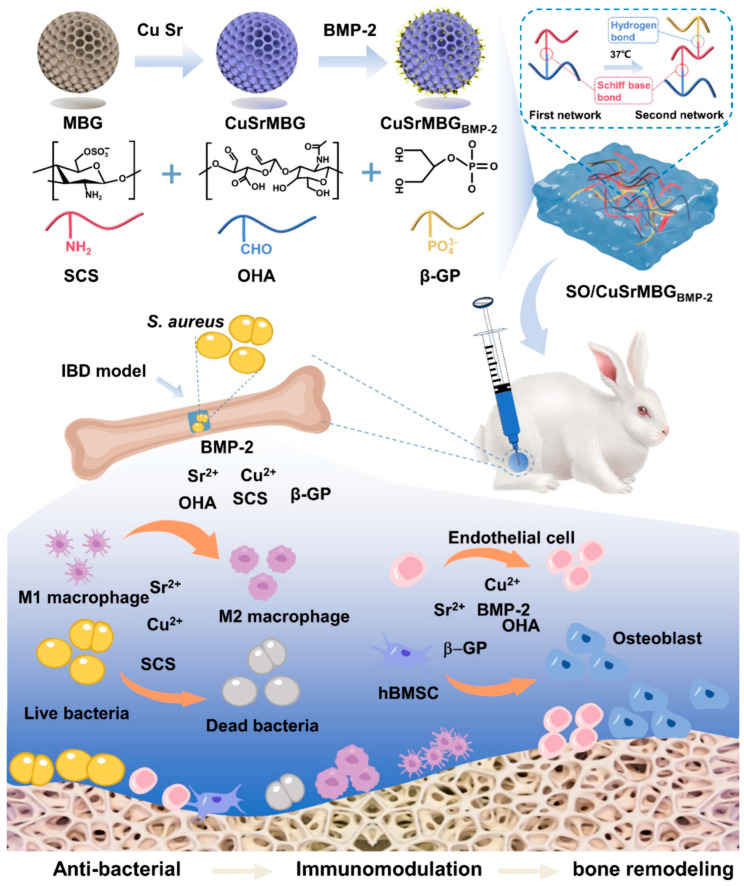
Schematic representation of the produced hydrogel. Reprinted with permission from Ref. [95]. Copyright 2024 Elsevier.

Wang et al. [95] produced hydrogels based on sulfated chitosan and oxidized hyaluronic acid-encapsulated mesoporous bioactive glass doped with copper (Cu) and strontium (Sr) (CuSrMBG) and loaded with bone morphogenetic protein-2 (BMP-2) (CuSrMBG_BMP-2_) (Figure 11). The hydrogel without CuSrMBG_BMP-2_ was designated as SO; the hydrogel with CuSrMBG was designated as SO/CuSrMBG. The in vitro and in vivo studies were performed using four different compositions: control, SO, SO/CuSrMBG, and SO/CuSrMBG_BMP-2_ hydrogels, respectively. Cell migration studies were performed using human umbilical vein endothelial cells (HUVECs) with SO/CuSrMBG and SO/CuSrMBG_BMP-2_ hydrogels, which demonstrated enhanced migration rates and promoted vascularization compared to the SO and control groups. Osteogenic differentiation was assessed in human bone marrow mesenchymal stem cells (hBMSCs). Comparing the control with the SO group, there were no differences between the two groups, showing the limited capacity of SO to promote ALP expression. Between the SO, SO/CuSrMBG, and SO/CuSrMBG_BMP-2_, the ALP expression was significantly higher in the last two groups, demonstrating the role of CuSrMBG in promoting ALP expression. However, the expression was even higher in the SO/CuSrMBG_BMP-2_ because of the addition of BMP-2, a bone growth factor that promotes bone formation.

In vivo studies in an infected rat bone defect model showed better results in the SO/CuSrMBG and SO/CuSrMBG_BMP-2_ groups, with good antibacterial activity against *S. aureus* and regenerative capacity with the decrease in the bone defect site compared to the other groups. Nevertheless, it was observed that new bone formation and osseointegration were higher in the SO/CuSrMBG_BMP-2_ group, demonstrating their potential in promoting bone regeneration [95].

Hia et al. proposed hydrogels composed of chitosan and poly(ethylene glycol)diacrylate (PEGDA) with copper-doped mesoporous silica nanospheres (Cu-MsNs). The NP concentrations added were 25, 50, 100, 150, and 200 µg/mL. Hydrogels with just PEGDA were used as a control. All the hydrogels presented good MC3T3-E1 cell viability; however, after 7 days of culture, the hydrogels with Cu-MsNs concentrations superior to 150 µg/mL showed a reduction in cell viability percentage. The hydrogels with a Cu-MsNs concentration of 100 µg/mL were the ones with a higher cell viability after 7 days in culture. In general, the hydrogels presented higher mechanical stability, cell viability, biodegradability, and osteogenic differentiation [96].

Cellulose is a natural polymer produced in plants or by bacteria [98] that is non-toxic, allowing it to be used in humans [99]. However, it is a natural polymer that requires chemical modification due to its high content of hydroxyl groups, unlike other natural polymers [99]. As it is produced in plants and bacteria, it exhibits intrinsic biocompatibility, biodegradability, and non-toxicity [98].

Titanium oxide NPs have been used in bone implants due to their mechanical properties, biocompatibility, low cytotoxicity, good permeability, and body fluid stability. Their structure causes positive effects in molecular response and osseointegration, stimulating bone formation. It has been suggested that it is material that can carry cells in its structure, where its properties can enhance cell viability [97,100].

Kasi et al. [97] prepared hydrogels based on chitosan, polyvinyl alcohol (PVA), microcrystalline cellulose (MC), and titanium dioxide NPs (TiO_2_NPs) with concentrations of 0.05, 0.1, 0.5, and 1%. MG-63 cells’ adhesion was higher in the hydrogels with a 1% concentration of TiO_2_NPs, compared to the other groups. These composite hydrogels allowed cell adhesion, spreading, and osteoblast differentiation, making it possible to be applied in bone regeneration applications.

Lastly, Rubina et al. [101] suggested ε-polylysine and hyaluronic acid hydrogels for bone regeneration. Sr-substituted hydroxyapatite nanoparticles (Sr-HAp) with concentrations of 0, 40, 50, and 60 wt% Sr-HAp were incorporated in the system. For ALP expression quantification and cell proliferation, two different types of cells, MG-63 and MC3T3-E1, were used. For MC3T3-E1, the ALP expression values decreased when increasing the percentage of Sr-HAp; the samples formed with 0% Sr-HAp were the ones that showed higher ALP expression. In the MG-63 cells, the samples with 40% and 50% Sr-HAp had higher ALP values than 0% and 60% Sr-HAp, indicating that concentrations higher than 60% and lower than 40% reduce osteogenic differentiation.

Regarding cell proliferation, the results were similar in both cell types. The hydrogels with 0% Sr-HAp were the ones that allowed for higher cell proliferation. However, after 5 days in culture, their values were lower than the control group. These results indicate that even though the NPs used stimulate osteogenic differentiation, they do not support cell proliferation [101].

Table 1 summarizes the main composite hydrogels filled with distinct nanoparticles studied for bone regeneration applications and their features.

## 6. Conclusions and Future Perspectives

Bone is a vital organ that provides structural support, protects essential organs, and maintains mineral homeostasis. It can self-regenerate in cases of minor fractures; however, more severe injuries often require medical intervention. This typically involves the use of bone grafts: autografts or allografts. While these materials offer numerous advantages, such as osseointegration, osteoinductivity, and osteoconductivity, they also present some disadvantages, primarily rejection, antibacterial infections, patient discomfort, pain, and the potential need for multiple surgeries to complete treatment. Due to these drawbacks, tissue engineering has emerged as a promising approach to develop safer materials for treating bone fractures utilizing biomaterials. Among various types of biomaterials, hydrogels have gained popularity because they allow for the easy production of scaffolds using different polymers tailored to specific applications. There are several types of natural polymers capable of being used in the development of hydrogels for bone regeneration. These polymers will help the cell proliferation process, angiogenesis, and the new bone formation.

Nevertheless, the use of hydrogels alone may present challenges related to mechanical strength and infection control. In recent years, the incorporation of nanoparticles into hydrogel systems has shown great potential to overcome these limitations. Nanoparticles can provide antimicrobial properties and enhanced mechanical performance. Their incorporation into hydrogels may reduce the risk that is typically associated with free NPs in biological systems. The resulting NP–hydrogel composites represent an emerging class of multifunctional biomaterials capable of promoting bone regeneration, improving wound healing, and minimizing infection. Furthermore, these systems can mimic the ECM, enhance mechanical strength, improve wound treatment, reduce the risk of infections, and stimulate tissue regeneration.

Although progress has been made, further investigation is required to achieve the full clinical potential of NP–hydrogel systems. Future efforts should focus on the development of more efficient, minimally invasive delivery methods, such as injectable formulations, which enhance patient comfort, reduce the risk of infections, and improve treatment efficacy. Furthermore, the development of bioadhesive hydrogels combined with NPs offers a promising strategy to improve fixation at defect sites, strengthen interfacial bonding with native bone tissue, and potentially eliminate the need for additional fixation hardware.

Advancements in the design and functionalization of NP–hydrogel composites could enable the development of next-generation therapies that provide safer and more effective treatments for complex bone injuries and regenerative medicine.

## Figures and Tables

**Table 1:** Composite hydrogels used in bone regeneration.

Composite	Features	Reference
Collagen–chitosan + selenium NPs	Increase in cell proliferation and osteogenic differentiation of MSCs	[88]
Gelatin + gold NPs	Osteogenic differentiation ADSC mineralization In vivo new bone formation	[81]
Chitosan/collagen + carboxylated single-walled carbon nanotubes	Increase in MC3T3-E1 cell proliferation Augmentation of osteogenic markers expression, calcium, and ALP	[90]
Alginate + selenium doped with biphasic calcium phosphate NPs + retinoic acid	Bioactive hydrogel with formation of HAp crystals Increase MC3T3-E1 cell viability and proliferation	[74]
PVA + chitosan + sodium alginate + carbon nanotubes or graphene nanoplatelets	Augmentation of MG-63 cell viability Swelling and degradation are favorable for bone tissue engineering	[93]
Gellan gum + nanohydroxyapatite + magnesium sulfate	Increase cell viability and proliferation of BMSCs Good cell adhesion Increase in the expression of osteogenic genes Osteogenic differentiation In vivo stimulated vascularization and new bone formation	[84]
Pluronic grafted silk fibroin hydrogel + hydroxyapatite NPs	Osteogenic promotion Cytocompatibility and cell proliferation of MG-63	[67]
Gelatin (catechol) + hydroxyapatite NPs	Cytocompatibility to MC3T3-E1 cells In vivo bone formation	[63]
Chitosan (sulfated) + oxidized hyaluronic acid + CuSrBG mesoporous bioactive glass dopes	Promotes osteogenesis and osteogenic differentiation of hBMSCs Increase in hBMSC viability In vivo bone regeneration and antibacterial activity against *S.aureus*	[95]
Silk fibroin/nanohydroxyapatite + silver NPs + gold NPs	Increase in MG-63 cell viability Allowed osteoblast adhesion and spreading	[65]
Chitosan (modified) + MgO NPs	In vitro mineralization Increase in osteogenic differentiation of MC3T3-E1 In vivo bone formation	[78]
Hyaluronic acid + biphosphate–magnesium NPs	Increase in cell adhesion and spreading of hMSCs Promoted mineralization and osteogenic differentiation In situ bone regeneration In vivo bone formation	[70]
Alginate + nanohydroxyapatite	In vitro osteoblast proliferation of hMSCs Ex vivo studies showed collagenase deposition and trabecular bone formation	[64]
Alginate–gelatin + fragmented nanofiber of polycaprolactone + bioactive glass NPs	Supported cell growth and proliferation of MG-63 Allowed cell adhesion	[86]
Chitosan phosphocreatine (functionalized) + MgO NPs	Increased cell proliferation of MC3T3-E1 Osteogenic differentiation Elevated expression of osteogenic genes In vivo bone regeneration	[75]
Poly (ethylene glycol) diacrylate (PEGDA) + chitosan + copper-doped mesoporous silica nanospheres	Elevated mechanical stability Increase MC3TE-E1 cell viability Osteogenic differentiation	[96]
Alginate–gelatin + silver NPs	Increase the cell viability of SHEDs Promotes osteogenic differentiation, with an increase in ALP activity	[87]
Fibrin + graphene oxide NPs	Enhanced cell viability at concentrations of 40 mg/mL Increased ALP activity and osteogenic differentiation In vivo bone healing	[71]
Silk fibroin + hydroxyapatite NPs	Increase in the ALP activity	[62]
Silk fibroin + silica NPs	Enhanced cell adhesion and proliferation of MC3T3-E1 In vitro osteogenic differentiation In vivo bone formation in cranial fractures	[67]
Collagen + strontium-containing nanosized mesoporous bioglass NPs	Osteogenic differentiation Viable to MG-63 cells Allow cell adhesion to the hydrogel structure	[55]
Collagen + strontium-enriched mesoporous bioglass or rod-like hydroxyapatite	Increase in PBMC viability Increased values of ALP activity	[56]
Chitosan + cellulose hydrogels + titanium dioxide NPs	Allowed cell adhesion, spreading, and MG-63 osteoblast differentiation	[97]
ε-polylysine + hyaluronic acid + Sr-substituted hydroxyapatite NPs	Osteogenic differentiation Increase the cell proliferation of MC3T3-E1 and MG-63	[101]
Gellan gum + bioglass nanoparticles	Bioactive hydrogels with the formation of HAp crystals Antibacterial activity against MRSA Cytocompatibility and supported MG-63 cell growth	[85]

## Data Availability

No new data were created or analyzed in this study. Data sharing is not applicable to this article.

## Abbreviations

The following abbreviations are used in this manuscript:

Ac-BP	Biphosphate nanoparticles
AgNPs	Silver nanoparticles
Alg	Alginate
ALP	Alkaline phosphatase
AuNPs	Gold nanoparticles
BGNPs	Bioglass nanoparticles
BMPs	Bone morphogenetic proteins
BTE	Bone tissue engineering
Chi	Chitosan
CNTs	Carbon nanotubes
Coll	Collagen
COOH-SWCNTs	Carboxylated single-walled carbon nanotubes
CTGF	Connective tissue growth factor
Cu-MsNs	Copper-doped mesoporous silica nanospheres
ECM	Extracellular matrix
FGF	Fibroblast growth factor
GG	Gellan gum
GNPs	Graphene nanoplatelets
GO	Graphene oxide nanoparticles
hMSCs	Human mesenchymal stem cells
MBG	Mesoporous bioglass nanoparticles
MC	Microcrystalline cellulose
MeHA	Methacrylated hyaluronic acid
MgONPs	Magnesium oxide nanoparticles
MSCs	Mesenchymal stem cells
nHA	Hydroxyapatite
NPs	Nanoparticles
PDGF	Platelet-derived growth factor
PEGDA	Poly(ethylene glycol)diacrylate
PVA	Polyvinyl alcohol
Se-BCP	Selenium biphasic calcium NPs
SF	Silk fibroin
SiNPs	Silica nanoparticles
TGF-beta	Transforming growth factor beta
TiO2NPs	Titanium dioxide NPs
VEGF	Vascular endothelial growth factor

## References

[1] Feng X. (2009). Chemical and Biochemical Basis of Cell-Bone Matrix Interaction in Health and Disease. Curr. Chem. Biol..

[2] Šromová V., Sobola D., Kaspar P. (2023). A Brief Review of Bone Cell Function and Importance. Cells.

[3] Koushik T.M., Miller C.M., Antunes E. (2023). Bone Tissue Engineering Scaffolds: Function of Multi-Material Hierarchically Structured Scaffolds. Adv. Healthc. Mater..

[4] Guillén-Carvajal K., Valdez-Salas B., Beltrán-Partida E., Salomón-Carlos J., Cheng N. (2023). Chitosan, Gelatin, and Collagen Hydrogels for Bone Regeneration. Polymers.

[5] Liu J., Yang L., Liu K., Gao F. (2023). Hydrogel scaffolds in bone regeneration: Their promising roles in angiogenesis. Front. Pharmacol..

[6] Florencio-Silva R., Sasso G.R.D.S., Sasso-Cerri E., Simões M.J., Cerri P.S. (2015). Biology of Bone Tissue: Structure, Function, and Factors That Influence Bone Cells. Biomed. Res. Int..

[7] Bai X., Gao M., Syed S., Zhuang J., Xu X., Zhang X.Q. (2018). Bioactive hydrogels for bone regeneration. Bioact Mater..

[8] Walmsley G.G., Ransom R.C., Zielins E.R., Leavitt T., Flacco J.S., Hu M.S., Lee A.S., Longaker M.T., Wan D.C. (2016). Stem Cells in Bone Regeneration. Stem Cell Rev. Rep..

[9] ElHawary H., Baradaran A., Abi-Rafeh J., Vorstenbosch J., Xu L., Efanov J.I. (2021). Bone Healing and Inflammation: Principles of Fracture and Repair. Semin. Plast. Surg..

[10] Steppe L., Megafu M., Tschaffon-Müller M.E.A., Ignatius A., Haffner-Luntzer M. (2023). Fracture healing research: Recent insights. Bone Rep..

[11] Beederman M., Lamplot J.D., Nan G., Wang J., Liu X., Yin L., Li R., Shui W., Zhang H., Kim S.H. (2013). BMP signaling in mesenchymal stem cell differentiation and bone formation. J. Biomed. Sci. Eng..

[12] Lee S.B., Lee H.J., Park J.B. (2023). Bone Morphogenetic Protein-9 Promotes Osteogenic Differentiation and Mineralization in Human Stem-Cell-Derived Spheroids. Medicina.

[13] Yue S., He H., Li B., Hou T. (2020). Hydrogel as a Biomaterial for Bone Tissue Engineering: A Review. Nanomaterials.

[14] Ho-Shui-Ling A., Bolander J., Rustom L.E., Johnson A.W., Luyten F.P., Picart C. (2018). Bone regeneration strategies: Engineered scaffolds, bioactive molecules and stem cells current stage and future perspectives. Biomaterials.

[15] Yousefi A.M. (2019). A review of calcium phosphate cements and acrylic bone cements as injectable materials for bone repair and implant fixation. J. Appl. Biomater. Funct. Mater..

[16] Bai L., Tao G., Feng M., Xie Y., Cai S., Peng S., Xiao J. (2023). Hydrogel Drug Delivery Systems for Bone Regeneration. Pharmaceutics.

[17] De Pace R., Molinari S., Mazzoni E., Perale G. (2025). Bone Regeneration: A Review of Current Treatment Strategies. J. Clin. Med..

[18] Ferraz M.P. (2023). Bone Grafts in Dental Medicine: An Overview of Autografts, Allografts and Synthetic Materials. Materials.

[19] Du J., Wang H., Zhong L., Wei S., Min X., Deng H., Zhang X., Zhong M., Huang Y. (2025). Bioactivity and biomedical applications of pomegranate peel extract: A comprehensive review. Front. Pharmacol..

[20] Correa S., Grosskopf A.K., Lopez Hernandez H., Chan D., Yu A.C., Stapleton L.M., Appel E.A. (2021). Translational Applications of Hydrogels. Chem. Rev..

[21] Deng X., Gould M., Ali M.A. (2022). A review of current advancements for wound healing: Biomaterial applications and medical devices. J. Biomed. Mater. Res. B Appl. Biomater..

[22] Cao H., Duan L., Zhang Y., Cao J., Zhang K. (2021). Current hydrogel advances in physicochemical and biological response-driven biomedical application diversity. Signal Transduct. Target Ther..

[23] Sun Y., Chen L.G., Fan X.M., Pang J.L. (2022). Ultrasound Responsive Smart Implantable Hydrogels for Targeted Delivery of Drugs: Reviewing Current Practices. Int. J. Nanomed..

[24] Jeong D., Jang S.Y., Roh S., Choi J.H., Seo I.J., Lee J.H., Kim J., Kwon I., Jung Y., Hwang J. (2024). Sprayable hydrogel with optical mRNA nanosensors for Real-Time monitoring and healing of diabetic wounds. Chem. Eng. J..

[25] Alonso J.M., Andrade del Olmo J., Perez Gonzalez R., Saez-Martinez V. (2021). Injectable Hydrogels: From Laboratory to Industrialization. Polymers.

[26] Wang H., Zhang L.M. (2024). Intelligent biobased hydrogels for diabetic wound healing: A review. Chem. Eng. J..

[27] Zhang Q., Liu Y., Yang G., Kong H., Guo L., Wei G. (2023). Recent advances in protein hydrogels: From design, structural and functional regulations to healthcare applications. Chem. Eng. J..

[28] García-García P., Reyes R., Pérez-Herrero E., Arnau M.R., Évora C., Delgado A. (2020). Alginate-hydrogel versus alginate-solid system. Efficacy in bone regeneration in osteoporosis. Mater. Sci. Eng. C.

[29] Gao Y., Zhang X., Zhou H. (2023). Biomimetic Hydrogel Applications and Challenges in Bone, Cartilage, and Nerve Repair. Pharmaceutics..

[30] Scheinpflug J., Pfeiffenberger M., Damerau A., Schwarz F., Textor M., Lang A., Schulze F. (2018). Journey into Bone Models: A Review. Genes.

[31] Li B., Li C., Yan Z., Yang X., Xiao W., Zhang D., Liu Z., Liao X. (2025). A review of self-healing hydrogels for bone repair and regeneration: Materials, mechanisms, and applications. Int. J. Biol. Macromol..

[32] Li W., Wu Y., Zhang X., Wu T., Huang K., Wang B., Liao J. (2023). Self-healing hydrogels for bone defect repair. RSC Adv..

[33] Choi H., Choi W.S., Jeong J.O. (2024). A Review of Advanced Hydrogel Applications for Tissue Engineering and Drug Delivery Systems as Biomaterials. Gels.

[34] Ho T.C., Chang C.C., Chan H.P., Chung T.W., Shu C.W., Chuang K.P., Duh T.-H., Yang M.-H., Tyan Y.-C. (2022). Hydrogels: Properties and Applications in Biomedicine. Molecules.

[35] Wen J., Cai D., Gao W., He R., Li Y., Zhou Y., Klein T., Xiao L., Xiao Y. (2023). Osteoimmunomodulatory Nanoparticles for Bone Regeneration. Nanomaterials.

[36] Ahmad N., Bukhari S.N.A., Hussain M.A., Ejaz H., Munir M.U., Amjad M.W. (2024). Nanoparticles incorporated hydrogels for delivery of antimicrobial agents: Developments and trends. RSC Adv..

[37] Lyons J.G., Plantz M.A., Hsu W.K., Hsu E.L., Minardi S. (2020). Nanostructured Biomaterials for Bone Regeneration. Front. Bioeng. Biotechnol..

[38] Kupikowska-Stobba B., Kasprzak M. (2021). Fabrication of nanoparticles for bone regeneration: New insight into applications of nanoemulsion technology. J. Mater. Chem. B.

[39] Hajiali H., Ouyang L., Llopis-Hernandez V., Dobre O., Rose F.R.A.J. (2021). Review of emerging nanotechnology in bone regeneration: Progress, challenges, and perspectives. Nanoscale.

[40] Saiz E., Zimmermann E.A., Lee J.S., Wegst U.G.K., Tomsia A.P. (2013). Perspectives on the role of nanotechnology in bone tissue engineering. Dental Mater..

[41] Liu X., Sun S., Wang N., Kang R., Xie L., Liu X. (2022). Therapeutic application of hydrogels for bone-related diseases. Front. Bioeng. Biotechnol..

[42] Hwang H.S., Lee C.S. (2024). Nanoclay-Composite Hydrogels for Bone Tissue Engineering. Gels.

[43] Omidian H., Chowdhury S.D. (2023). Advancements and Applications of Injectable Hydrogel Composites in Biomedical Research and Therapy. Gels.

[44] Kuang L., Ma X., Ma Y., Yao Y., Tariq M., Yuan Y., Changsheng L. (2019). Self-Assembled Injectable Nanocomposite Hydrogels Coordinated by in Situ Generated CaP Nanoparticles for Bone Regeneration. ACS Appl. Mater. Interfaces.

[45] Du C., Huang W. (2022). Progress and prospects of nanocomposite hydrogels in bone tissue engineering. Nanocomposites.

[46] Pablos J.L., Lozano D., Manzano M., Vallet-Regí M. (2024). Regenerative medicine: Hydrogels and mesoporous silica nanoparticles. Mater. Today Bio..

[47] Alshangiti D.M., El-damhougy T.K., Zaher A., Madani M., Mohamady ghobashy M. (2023). Revolutionizing biomedicine: Advancements, applications, and prospects of nanocomposite macromolecular carbohydrate-based hydrogel biomaterials: A review. RSC Adv..

[48] Ao Y., Zhang E., Liu Y., Yang L., Li J., Wang F. (2022). Advanced Hydrogels With Nanoparticle Inclusion for Cartilage Tissue Engineering. Front. Bioeng. Biotechnol..

[49] Xue J., Gurav N., Elsharkawy S., Deb S. (2024). Hydrogel Composite Magnetic Scaffolds: Toward Cell-Free In Situ Bone Tissue Engineering. ACS Appl. Bio. Mater..

[50] Suba Sri M., Usha R. (2025). An insightful overview on osteogenic potential of nano hydroxyapatite for bone regeneration. Cell Tissue Bank..

[51] Zhang G., Zhen C., Yang J., Wang J., Wang S., Fang Y., Shang P. (2024). Recent advances of nanoparticles on bone tissue engineering and bone cells. Nanoscale Adv..

[52] Gong T., Xie J., Liao J., Zhang T., Lin S., Lin Y. (2015). Nanomaterials and bone regeneration. Bone Res..

[53] Filippi M., Born G., Chaaban M., Scherberich A. (2020). Natural Polymeric Scaffolds in Bone Regeneration. Front. Bioeng. Biotechnol..

[54] Pourhajrezaei S., Abbas Z., Khalili M.A., Madineh H., Jooya H., Babaeizad A., Gross J.D., Samadi A. (2024). Bioactive polymers: A comprehensive review on bone grafting biomaterials. Int. J. Biol. Macromol..

[55] Montalbano G., Borciani G., Pontremoli C., Ciapetti G., Mattioli-Belmonte M., Fiorilli S., Vitale-Brovarone C. (2019). Development and Biocompatibility of Collagen-Based Composites Enriched with Nanoparticles of Strontium Containing Mesoporous Glass. Materials.

[56] Borciani G., Montalbano G., Melo P., Baldini N., Ciapetti G., Vitale-Brovarone C. (2021). Assessment of Collagen-Based Nanostructured Biomimetic Systems with a Co-Culture of Human Bone-Derived Cells. Cells.

[57] Naruphontjirakul P., Panpisut P., Patntirapong S. (2023). Zinc and Strontium-Substituted Bioactive Glass Nanoparticle/Alginate Composites Scaffold for Bone Regeneration. Int. J. Mol. Sci..

[58] Deshmukh K., Kovářík T., Křenek T., Docheva D., Stich T., Pola J. (2020). Recent advances and future perspectives of sol–gel derived porous bioactive glasses: A review. RSC Adv..

[59] Lee K.Z., Jeon J., Jiang B., Subramani S.V., Li J., Zhang F. (2023). Protein-Based Hydrogels and Their Biomedical Applications. Molecules.

[60] Wang Y., Yang Z., Chen X., Jiang X., Fu G. (2023). Silk fibroin hydrogel membranes prepared by a sequential cross-linking strategy for guided bone regeneration. J. Mech. Behav. Biomed. Mater..

[61] Amorim S., Dudik O., Soares da Costa D., Reis R.L., Silva T.H., Pires R.A. (2023). Fucoidan-Coated Silica Nanoparticles Promote the Differentiation of Human Mesenchymal Stem Cells into the Osteogenic Lineage. ACS Biomater. Sci. Eng..

[62] Ribeiro M., de Moraes M.A., Beppu M.M., Garcia M.P., Fernandes M.H., Monteiro F.J., Ferraz M.P. (2015). Development of silk fibroin/nanohydroxyapatite composite hydrogels for bone tissue engineering. Eur. Polym. J..

[63] Ma W., Chen H., Cheng S., Wu C., Wang L., Du M. (2023). Gelatin hydrogel reinforced with mussel-inspired polydopamine-functionalized nanohydroxyapatite for bone regeneration. Int. J. Biol. Macromol..

[64] Barros J., Ferraz M.P., Azeredo J., Fernandes M.H., Gomes P.S., Monteiro F.J. (2019). Alginate-nanohydroxyapatite hydrogel system: Optimizing the formulation for enhanced bone regeneration. Mater. Sci. Eng. C.

[65] Ribeiro M., Ferraz M.P., Monteiro F.J., Fernandes M.H., Beppu M.M., Mantione D., Sardon H. (2017). Antibacterial silk fibroin/nanohydroxyapatite hydrogels with silver and gold nanoparticles for bone regeneration. Nanomedicine.

[66] Yang D.H., Nah H., Lee D., Min S.J., Park S., An S.H., Wang J., He H., Choi K.-S., Ko W.-K. (2024). A review on gold nanoparticles as an innovative therapeutic cue in bone tissue engineering: Prospects and future clinical applications. Mater. Today Bio..

[67] Cheng Y., Cheng G., Xie C., Yin C., Dong X., Li Z., Zhou X., Wang Q., Deng H., Li Z. (2021). Biomimetic Silk Fibroin Hydrogels Strengthened by Silica Nanoparticles Distributed Nanofibers Facilitate Bone Repair. Adv. Healthc. Mater..

[68] Daneshvar A., Farokhi M., Bonakdar S., Vossoughi M. (2024). Synthesis and characterization of injectable thermosensitive hydrogel based on Pluronic-grafted silk fibroin copolymer containing hydroxyapatite nanoparticles as potential for bone tissue engineering. Int. J. Biol. Macromol..

[69] Hwang H.S., Lee C.S. (2023). Recent Progress in Hyaluronic-Acid-Based Hydrogels for Bone Tissue Engineering. Gels.

[70] Zhang K., Lin S., Feng Q., Dong C., Yang Y., Li G., Bian L. (2017). Nanocomposite hydrogels stabilized by self-assembled multivalent bisphosphonate-magnesium nanoparticles mediate sustained release of magnesium ion and promote in-situ bone regeneration. Acta Biomater..

[71] Pathmanapan S., Periyathambi P., Anandasadagopan S.K. (2020). Fibrin hydrogel incorporated with graphene oxide functionalized nanocomposite scaffolds for bone repair—In vitro and in vivo study. Nanomedicine.

[72] Brinkmann J., Malyaran H., Enezy-Ulbrich MAAl Jung S., Radermacher C., Buhl E.M., Pich A., Neuss S. (2023). Assessment of Fibrin-Based Hydrogels Containing a Fibrin-Binding Peptide to Tune Mechanical Properties and Cell Responses. Macromol. Mater. Eng..

[73] Govindarajan D., Saravanan S., Sudhakar S., Vimalraj S. (2024). Graphene: A Multifaceted Carbon-Based Material for Bone Tissue Engineering Applications. ACS Omega.

[74] Singhmar R., Son Y., Jo Y.J., Zo S., Min B.K., Sood A., Han S.S. (2024). Fabrication of alginate composite hydrogel encapsulated retinoic acid and nano Se doped biphasic CaP to augment in situ mineralization and osteoimmunomodulation for bone regeneration. Int. J. Biol. Macromol..

[75] Chen Y., Sheng W., Lin J., Fang C., Deng J., Zhang P., Zhou M., Liu P., Weng J., Yu F. (2022). Magnesium Oxide Nanoparticle Coordinated Phosphate-Functionalized Chitosan Injectable Hydrogel for Osteogenesis and Angiogenesis in Bone Regeneration. ACS Appl. Mater. Interfaces.

[76] Malaiappan S., Harris J. (2024). Osteogenic Potential of Magnesium Oxide Nanoparticles in Bone Regeneration: A Systematic Review. Cureus.

[77] Hosseini S.F., Galefi A., Hosseini S., Shaabani A., Farrokhi N., Jahanfar M., Nourany M., Homaeigohar S., Alipour A., Shahsavarani H. (2024). Magnesium oxide nanoparticle reinforced pumpkin-derived nanostructured cellulose scaffold for enhanced bone regeneration. Int. J. Biol. Macromol..

[78] Chen Y., Li C., Wang Z., Long J., Wang R., Zhao J., Tang W., Zhao Y., Qin L., Peng S. (2021). Self-assembled nanocomposite hydrogels enhanced by nanoparticles phosphonate-magnesium coordination for bone regeneration. Appl. Mater. Today.

[79] Paltanea G., Manescu (Paltanea) V., Antoniac I., Antoniac A., Nemoianu I.V., Robu A., Dura H. (2023). A Review of Biomimetic and Biodegradable Magnetic Scaffolds for Bone Tissue Engineering and Oncology. Int. J. Mol. Sci..

[80] Li J., Wang W., Li M., Song P., Lei H., Gui X., Zhou C., Liu L. (2021). Biomimetic Methacrylated Gelatin Hydrogel Loaded With Bone Marrow Mesenchymal Stem Cells for Bone Tissue Regeneration. Front. Bioeng. Biotechnol..

[81] Heo D.N., Ko W.K., Bae M.S., Lee J.B., Lee D.W., Byun W., Zhou C., Liu L. (2014). Enhanced bone regeneration with a gold nanoparticle–hydrogel complex. J. Mater. Chem. B.

[82] Wu S., Xiao R., Wu Y., Xu L. (2024). Advances in tissue engineering of gellan gum-based hydrogels. Carbohydr. Polym..

[83] Thangavelu M., Kim P.Y., Cho H., Song J.E., Park S., Bucciarelli A., Khang G. (2024). A Gellan Gum, Polyethylene Glycol, Hydroxyapatite Composite Scaffold with the Addition of Ginseng Derived Compound K with Possible Applications in Bone Regeneration. Gels.

[84] Liu H., Li K., Guo B., Yuan Y., Ruan Z., Long H., Zhu J., Zhu Y., Chen C. (2024). Engineering an injectable gellan gum-based hydrogel with osteogenesis and angiogenesis for bone regeneration. Tissue Cell.

[85] Douglas T.E.L., Piwowarczyk W., Pamula E., Liskova J., Schaubroeck D., Leeuwenburgh S.C.G., Brackman G., Balcaen L., Detsch R., Declercq H. (2014). Injectable self-gelling composites for bone tissue engineering based on gellan gum hydrogel enriched with different bioglasses. Biomed. Mater..

[86] Shahrebabaki K.E., Labbaf S., Karimzadeh F., Goli M., Mirhaj M. (2024). Alginate-gelatin based nanocomposite hydrogel scaffold incorporated with bioactive glass nanoparticles and fragmented nanofibers promote osteogenesis: From design to in vitro studies. Int. J. Biol. Macromol..

[87] Chavez-Granados P.A., Garcia-Contreras R., Reyes-Lopez C.A.S., Correa-Basurto J., Hernandez-Rojas I.E., Hernandez-Gomez G., Jurado C.A., Alhotan A. (2024). Green Synthesis of Silver Nanoparticles with Roasted Green Tea: Applications in Alginate–Gelatin Hydrogels for Bone Regeneration. Gels.

[88] Alajmi K., Hartford M., Roy N.S., Bhattacharya A., Kaity S., Cavanagh B.L., Roy S., Kaur K. (2024). Selenium nanoparticle-functionalized injectable chitosan/collagen hydrogels as a novel therapeutic strategy to enhance stem cell osteoblastic differentiation for bone regeneration. J. Mater. Chem. B.

[89] Karthik K.K., Cheriyan B.V., Rajeshkumar S., Gopalakrishnan M. (2024). A review on selenium nanoparticles and their biomedical applications. Biomed. Technol..

[90] Kaur K., Paiva S.S., Caffrey D., Cavanagh B.L., Murphy C.M. (2021). Injectable chitosan/collagen hydrogels nano-engineered with functionalized single wall carbon nanotubes for minimally invasive applications in bone. Mater. Sci. Eng. C.

[91] Chen Y., Li X. (2022). The utilization of carbon-based nanomaterials in bone tissue regeneration and engineering: Respective featured applications and future prospects. Med. Nov. Technol. Devices.

[92] Pei B., Wang W., Dunne N., Li X. (2019). Applications of Carbon Nanotubes in Bone Tissue Regeneration and Engineering: Superiority, Concerns, Current Advancements, and Prospects. Nanomaterials.

[93] Seifi S., Shamloo A., Barzoki A.K., Bakhtiari M.A., Zare S., Cheraghi F., Peyrovan A. (2024). Engineering biomimetic scaffolds for bone regeneration: Chitosan/alginate/polyvinyl alcohol-based double-network hydrogels with carbon nanomaterials. Carbohydr. Polym..

[94] Raslan A., Saenz del Burgo L., Ciriza J., Pedraz J.L. (2020). Graphene oxide and reduced graphene oxide-based scaffolds in regenerative medicine. Int. J. Pharm..

[95] Wang S., Lei H., Mi Y., Ma P., Fan D. (2024). Chitosan and hyaluronic acid based injectable dual network hydrogels—Mediating antimicrobial and inflammatory modulation to promote healing of infected bone defects. Int. J. Biol. Macromol..

[96] Hia E.M., Jang S.R., Maharjan B., Park J., Park C.H., Kim C.S. (2024). Construction of a PEGDA/chitosan hydrogel incorporating mineralized copper-doped mesoporous silica nanospheres for accelerated bone regeneration. Int. J. Biol. Macromol..

[97] Kasi P.B., Azar M.G., Dodda J.M., Bělský P., Kovářík T., Šlouf M., Dobrá J.K., Babuška V. (2023). Chitosan and cellulose-based composite hydrogels with embedded titanium dioxide nanoparticles as candidates for biomedical applications. Int. J. Biol. Macromol..

[98] Fu L.H., Qi C., Ma M.G., Wan P. (2019). Multifunctional cellulose-based hydrogels for biomedical applications. J. Mater. Chem. B.

[99] Kundu R., Mahada P., Chhirang B., Das B. (2022). Cellulose hydrogels: Green and sustainable soft biomaterials. Curr. Res. Green Sustain. Chem..

[100] Saber-Samandari S., Yekta H., Ahmadi S., Alamara K. (2018). The role of titanium dioxide on the morphology, microstructure, and bioactivity of grafted cellulose/hydroxyapatite nanocomposites for a potential application in bone repair. Int. J. Biol. Macromol..

[101] Rubina A., Sceglovs A., Ramata-Stunda A., Pugajeva I., Skadins I., Boyd A.R., Tumilovica A., Stipniece L., Salma-Ancane K. (2024). Injectable mineralized Sr-hydroxyapatite nanoparticles-loaded ɛ-polylysine-hyaluronic acid composite hydrogels for bone regeneration. Int. J. Biol. Macromol..

